# Advances in Microbial Solutions for Sustainable Agriculture

**DOI:** 10.3390/plants15193027

**Published:** 2026-10-03

**Authors:** Ivica Dimkić

**Affiliations:** Department of Biochemistry and Molecular Biology, Faculty of Biology, University of Belgrade, Studentski trg 16, 11158 Belgrade, Serbia; ivicad@bio.bg.ac.rs

This Special Issue, “Advances in Microbial Solutions for Sustainable Agriculture,” comprises ten high-quality contributions that collectively reflect the current state of the art in plant-associated microbiome research. Covering topics from rhizosphere ecology and microbial community dynamics to biological control, plant growth promotion, and microbiome-based agricultural biotechnologies, the collection highlights both fundamental discoveries and their translation into sustainable crop management practices [1,2,3,4,5,6,7,8,9,10]. The environmental and agronomic costs associated with the intensive use of chemical fertilizers and pesticides have increased interest in plant-associated microorganisms as components of sustainable crop-production systems. The contributions to this Special Issue illustrate the diversity of processes through which microorganisms affect plant health, including nutrient mobilization, rhizosphere community assembly, plant growth promotion, induced resistance, pathogen suppression, and adaptation to environmental stress. The studies encompass agricultural and natural ecosystems and employ approaches ranging from microbial inoculation and soil amendment experiments to metagenomics, microbiome profiling, biochemical characterization, and plant defense analysis. Collectively, they show that microbiome-based agricultural strategies must consider microbial functions within their ecological and environmental contexts. Single strains, microbial consortia, organic amendments, and microbial metabolites can contribute to crop productivity and protection, but their effects depend on the plant host, soil characteristics, developmental stage, formulation, and field environment. This editorial summarizes the principal advances presented in the Special Issue and identifies priorities for translating plant-microbe research into reliable biofertilizers, biostimulants, biopesticides, and integrated crop-management strategies.

## 1. Introduction

Increasing crop productivity while reducing the environmental burden of agriculture is a defining challenge for contemporary plant science. Synthetic fertilizers and pesticides have contributed substantially to global food production, but their intensive or poorly managed use can promote nutrient losses, environmental contamination, soil degradation, and the selection of resistant plant pathogens. Biological approaches based on plant-associated microorganisms offer an opportunity to complement conventional inputs while supporting crop productivity and ecosystem functions. The plant microbiome comprises microorganisms inhabiting the rhizosphere, root endosphere, phyllosphere, seeds, and surrounding soil. Its composition and activity are influenced by the plant genotype, developmental stage, soil characteristics, climate, management practices, and interactions among microbial populations [11,12,13]. Beneficial microorganisms may improve plant performance through nitrogen fixation, phosphate solubilization, siderophore production, phytohormone modulation, improved water and nutrient acquisition, and protection against environmental stress. They may also suppress pathogens through competition, antibiosis, lytic enzymes, immune activation, and changes in the wider microbial community [11,14,15].

The contributions assembled in this Special Issue illustrate the transition of plant-microbiome research from descriptive community surveys toward functional investigation and agricultural application. The studies examine how soil properties and amendments restructure microbial communities, how bacterial and algal inoculants influence crop performance, how microbial metabolites regulate plant immunity, and how biological formulations perform under greenhouse and field conditions [1,2,3,4,5,6,7,8,9,10]. Collectively, these contributions demonstrate the potential of microbiome-assisted agriculture while emphasizing that the success of microbial interventions remains strongly dependent on environmental and biological context.

## 2. Soil Environment and Microbiome Function

Soil physico-chemical properties are among the principal determinants of rhizosphere microbiome assembly and activity. Lan et al. compared peanut growth and bacterial communities in neutral, acidic, and saline-alkali soils [1]. Saline-alkali soil caused the strongest reductions in plant growth and yield and was associated with lower available phosphorus, reduced soil enzyme activities, decreased microbial richness, and lower abundances of potentially beneficial genera, including *Bradyrhizobium* and *Streptomyces*. Metagenomic analysis also indicated reductions in microbial pathways related to soil remediation, fertility improvement, and stress adaptation. These results demonstrate that unfavorable soils influence plants through more than direct physico-chemical stress. Changes in pH, salinity, and nutrient availability can restructure microbial communities and weaken biological processes supporting nutrient cycling and plant resilience. The study therefore highlights the value of combining soil chemistry, plant phenotyping, enzyme assays, and functional microbiome analysis when evaluating low-productivity agricultural soils.

Li et al. investigated the effects of corn-stover biochar in continuously cropped tomato soil [5]. Biochar altered nitrogen and phosphorus transformations, bacterial community diversity, and soil enzyme activities. However, these effects depended on the application rate, with the lowest tested rate producing the most favorable overall enzyme response. This finding shows that increasing amendment inputs does not necessarily produce proportional biological benefits. Biochar application should therefore be optimized according to soil characteristics, crop requirements, microbial responses, and possible effects on nutrient availability.

At a broader ecosystem scale, Wen et al. examined how microorganisms and soil environmental factors jointly determine ecosystem multifunctionality in semi-arid wetlands [9]. Riverine and lacustrine wetlands differed in microbial diversity, soil properties, enzyme activities, plant communities, and multifunctionality. Microbial diversity was more directly associated with multifunctionality in riverine wetlands, whereas salinity and soil moisture exerted stronger environmental filtering in lacustrine wetlands. Although this study concerns natural wetlands rather than crop fields, its conclusions are relevant to agricultural microbiome management. Microbial functions cannot be interpreted separately from their habitat. Environmental conditions determine which microorganisms can establish, how microbial networks are structured, and whether potentially beneficial functions are expressed. This ecological dependency represents one of the major challenges in transferring microorganisms selected under controlled conditions into agricultural soils [12,13].

## 3. Microbial Inoculants and Plant Growth Promotion

Several contributions explore the practical application of beneficial microorganisms. Kruščić et al. evaluated a formulation containing *Bacillus subtilis* and *Microbacterium* sp., poultry manure, and their combined use in a maize cropping system [2]. The treatments altered rhizosphere and root-associated bacterial communities, but their effects changed during plant development. Poultry manure initially enriched several potentially beneficial taxa, whereas the combined treatments influenced bacterial diversity and plant performance. The temporal character of these responses is particularly important. Microbial inoculation may produce an early shift in community composition that weakens or disappears as plant development, root exudation, nutrient availability, and environmental conditions change. Consequently, inoculant performance should be monitored throughout the crop cycle rather than inferred from a single sampling point [11]. The interaction between introduced organisms, organic amendments, and resident microbiota must also be considered when developing combined biological products.

Anđelković et al. tested ten soil bacterial strains on maize, tomato, cucumber, and pepper under greenhouse conditions [3]. Different strains produced the strongest effects on different plant hosts. *Bacillus safensis* SCF6 was particularly effective across all tested plants, whereas *Microbacterium testaceum*, *Bacillus mojavensis*, and *Pseudomonas putida* produced notable responses in pepper, tomato, and cucumber, respectively. Several isolates also exhibited antifungal activity. These results underline the importance of strain–host compatibility. The detection of growth-promoting traits under laboratory conditions does not guarantee consistent performance across crops. Colonization capacity, root-exudate chemistry, plant immune responses, indigenous microbial competition, and the physical soil environment can all influence inoculant effectiveness. Candidate strains should therefore be tested on their intended crop and under conditions representative of the planned agricultural application [14,16].

Tajti et al. examined an inter-kingdom co-culture consisting of *Chlorella* sp. and *Kocuria rhizophila* in tomato [7]. The combined treatment improved plant biomass, photosynthetic performance, fruit characteristics, and several yield-associated parameters more effectively than either organism applied individually. These effects were associated partly with enhanced production of extracellular polymeric substances by the alga during co-cultivation. This work demonstrates the potential of designing biological products around functional complementarity. Algae and bacteria may provide different but mutually supportive activities, including nutrient mobilization, production of signaling compounds, improvement of the root environment, and protection from stress. Inter-kingdom consortia may therefore offer functions that are difficult to obtain from individual strains.

Functional cooperation is also central to the review by Yoshida and Teaumroong on *Bacillus velezensis* S141 in soybean [8]. This bacterium possesses traits related to phytohormone production, drought adaptation, transformation of soybean isoflavones, and interactions with other rhizosphere microorganisms. Its combined application with *Bradyrhizobium* can improve nodulation, nitrogen fixation, and soybean yield. Such findings support the rational construction of microbial consortia, although their ecological stability and reproducibility remain important limitations [17,18].

## 4. Biological Control and Plant Defense

Beneficial microorganisms can suppress plant diseases through direct antagonism, resource competition, and activation of plant defense pathways. Ding et al. reviewed cyclic lipopeptides produced by *Bacillus*, *Pseudomonas*, *Paenibacillus*, *Burkholderia*, *Serratia*, and *Streptomyces* species [4]. Surfactins, fengycins, iturins, massetolides, and other cyclic lipopeptides may act both as antimicrobial compounds and as elicitors of induced systemic resistance. The biological effects of these molecules depend on their chemical structures, concentrations, producing strains, target plants, pathogens, and application methods. Microbial metabolites should therefore not be considered universally effective elicitors. Their use requires crop- and pathogen-specific optimization, as well as evaluation of possible phytotoxicity, environmental persistence, and interactions with non-target organisms. The translation of cyclic lipopeptides into biological crop-protection products will require improvement of fermentation yields, downstream recovery, formulation, storage stability, delivery, and field persistence. These challenges are representative of the wider microbial biopesticide sector, where strong in vitro activity does not always produce reliable field protection [16,19].

Palermo et al. examined interactions among sweet basil, *Bacillus amyloliquefaciens* GB03, and aphid *Acyrthosiphon pisum* [6]. Bacterial inoculation modified essential oils, volatile organic compounds, phytohormones, and glandular trichome density upon aphid herbivory. However, aphids preferred inoculated plants, potentially because of changes in volatile emissions. This result demonstrates the complexity of plant–microbe–insect interactions. Activation of biochemical or structural defense responses does not necessarily translate directly into pest deterrence. Microbial inoculants may simultaneously affect plant physiology, herbivore behavior, natural enemies, and volatile signaling. Evaluations based only on defense markers or growth parameters may therefore overlook ecologically important outcomes.

Ruiz et al. evaluated *Pseudomonas protegens*-based formulations for the management of grapevine powdery mildew and gray mold under field conditions [10]. The formulations activated defense-related genes, including *pr1*, *pr2*, and *pr10*, and moderately reduced disease severity. Although their effects were lower than those of conventional treatments, the results indicate potential for their use as complementary components of integrated disease-management programs. Biological agents may be particularly valuable when combined with resistant cultivars, cultural practices, disease forecasting, and reduced chemical applications. Their contribution should not necessarily be evaluated according to whether they completely replace fungicides but according to whether they reduce chemical dependence, delay resistance development, and maintain disease control within economically acceptable limits.

## 5. From Microbiome Characterization to Agricultural Application

The contributions to this Special Issue support a broader transition from cataloging microbial taxa toward determining microbial functions and designing predictable biological interventions. Sequencing-based analyses can identify associations between microbial groups, soil properties, and plant performance, but taxonomic correlations alone do not establish causality. Metagenomics, transcriptomics, metabolomics, microbial isolation, enzyme assays, synthetic-community experiments, and plant phenotyping should be combined to determine which microorganisms are active and which mechanisms contribute to agronomic outcomes [12,13,20].

Synthetic or simplified microbial communities offer a promising approach because they can combine nutrient mobilization, stress protection, pathogen suppression, and niche occupation within a defined formulation [17,18]. Nevertheless, increasing consortium complexity does not automatically improve performance. Consortium members must be mutually compatible, able to colonize the intended plant, and sufficiently stable under fluctuating field conditions. Microbiome engineering can also operate through the plant host. Selection or breeding for plant traits that favor beneficial microbiota may provide a more persistent strategy than repeated inoculation alone [16]. Root architecture, exudate composition, immune regulation, and nutrient transport can influence microbiome recruitment and may be incorporated into future crop-improvement programs.

Commercial translation requires equal attention to microbiology and application technology. Selected organisms must survive production, formulation, storage, transportation, and delivery. Products must also show an acceptable shelf life, biosafety, compatibility with existing farming practices, and reproducibility across soils, climates, and growing seasons [11,14,19]. Regulatory assessment and quality control will be essential for distinguishing effective, well-characterized products from formulations supported only by laboratory screening.

## 6. Conclusions and Future Perspectives

The articles in this Special Issue demonstrate that plant-associated microorganisms are central participants in crop nutrition, stress adaptation, soil functioning, and biological control. They also show that beneficial outcomes are highly context dependent. Soil conditions restructure microbial communities, different crops respond differently to the same bacterial strains, microbial partnerships may provide complementary functions, and defense activation may generate unexpected ecological effects. Future studies should prioritize longitudinal and multi-location field trials, functional validation of microbiome data, and the development of indicators capable of predicting inoculant performance. Greater attention should be given to microbial consortia, inter-kingdom interactions, host-mediated microbiome recruitment, formulation technology, and integration with organic amendments and precision crop management. Plant microbiome research is moving toward a framework in which plants, microorganisms, soils, pathogens, pests, and agricultural practices are considered components of a connected biological system. By combining mechanistic research with ecological validation and scalable product development, plant-associated microorganisms may become increasingly reliable tools for productive, resilient, and environmentally sustainable agriculture.
